# Distinct Bleaching Resilience of Photosynthetic Plastid-Bearing Mollusks Under Thermal Stress and High CO_2_ Conditions

**DOI:** 10.3389/fphys.2018.01675

**Published:** 2018-11-30

**Authors:** Gisela Dionísio, Filipa Faleiro, Regina Bispo, Ana Rita Lopes, Sónia Cruz, José Ricardo Paula, Tiago Repolho, Ricardo Calado, Rui Rosa

**Affiliations:** ^1^MARE – Marine and Environmental Sciences Centre, Laboratório Marítimo da Guia – Faculdade de Ciências da Universidade de Lisboa, Cascais, Portugal; ^2^Departamento de Biologia & CESAM & ECOMARE, Universidade de Aveiro, Aveiro, Portugal; ^3^Naturalist Science & Tourism, Horta, Portugal; ^4^Departamento de Matemática, Centro de Matemática e Aplicações, Faculdade de Ciências e Tecnologia, Universidade Nova de Lisboa, Costa de Caparica, Portugal

**Keywords:** climate change, kleptoplasty, bleaching, photobiology, oxidative stress, metabolism, mollusk-plastid association

## Abstract

The impact of temperature on photo-symbiotic relationships has been highly studied in the tropical reef-forming corals but overlooked in less charismatic groups such as solar-powered sacoglossan sea slugs. These organisms display one of the most puzzling symbiotic features observed in the animal kingdom, i.e., their mollusk-plastid association, which enables them to retain photosynthetic active chloroplasts (i.e., kleptoplasts) retrieved from their algae feed sources. Here we analyze the impact of thermal stress (+4°C) and high *p*CO_2_ conditions (ΔpH = 0.4) in survival, photophysiology (i.e., bleaching, photosynthetic efficiency, and metabolism) and stress defense mechanisms (i.e., heat shock and antioxidant response) of solar-powered sacoglossan sea slugs, from tropical (*Elysia crispata*) and temperate (*E. viridis*) environments. High temperature was the main factor affecting the survival of both species, while pH only affected the survival of the temperate model. The photobiology of *E. viridis* remained stable under the combined scenario, while photoinhibition was observed for *E. crispata* under high temperature and high *p*CO_2_. In fact, bleaching was observed within all tropical specimens exposed to warming (but not in the temperate ones), which constitutes the first report where the incidence of bleaching in tropical animals hosting photosynthetic symbionts, other than corals, occurs. Yet, the expulsion of kleptoplasts by the tropical sea slug, allied with metabolic depression, constituted a physiological response that did not imply signs of vulnerability (i.e., mortality) in the host itself. Although the temperate species revealed greater heat shock and antioxidant enzyme response to environmental stress, we argue that the tropical (stenotherm) sea slug species may display a greater scope for acclimatization than the temperate (eurytherm) sea slug. *E. crispata* may exhibit increased capacity for phenotypic plasticity by increasing fitness in a much narrower thermal niche (minimizing maintenance costs), which ultimately may allow to face severe environmental conditions more effectively than its temperate generalist counterpart (*E. viridis)*.

## Introduction

Kleptoplasty is an exciting research topic once it represents a unique naturally occurring biological condition, where chloroplasts can be found intra-cellularly within organisms phylogenetically distant from the algae host in which they evolved (Serôdio et al., 2014). This photosynthetic association results from the maintenance of photosynthetically competent chloroplasts, often termed “kleptoplasts,” sequestered from algae that remain structurally intact and temporarily functional (Rumpho et al., 2006; Pierce and Curtis, 2012). Symbiont photosynthesis plays a major role in the nutrient acquisition of these associations (Tremblay et al., 2013). Kleptoplasty may be especially valuable within environments where other nutrient sources remain in short supply (Venn et al., 2008) or even as a mean to overcome periods when algae feed is either absent (e.g., during winter months) or calcifying (e.g., in the case of *Elysia timida* see Casalduero and Muniain, 2008).

Over the last decades, anthropogenic pressures on the planet have resulted in an unprecedented increase in atmospheric carbon dioxide (CO_2_) concentration. As a result, atmospheric CO_2_ is increasingly being dissolved in the ocean, causing a risein its acidity, thus leading to an upsurge of the ocean acidification phenomena. As such, a decrease of 0.1 units in surface water pH was observed over the last decades, with projections indicating a further decrease between 0.14 and 0.42 units, by the end of the 21st century (Pörtner et al., 2014). Another result of the escalation of atmospheric partial pressure of carbon dioxide (*p*CO_2_) is the increase in global temperatures, with future projections estimating an increase of sea surface temperature (SST) of 3–4°C, by the end of the century (IPCC, 2013). Such future changes in ocean’s physical and chemical properties are expected to pose, to a more or less extent, biological restraints over marine biota (Kroeker et al., 2013). In this matter, tropical organisms are expected to be more vulnerable when faced upon future warming and acidification conditions, in comparison to all of those with temperate environments (Nilsson et al., 2009; Rosa et al., 2014).

Considering the impact of climate change on sun-powered animals, most studies have been focused on the tropical reef-forming corals and their symbiotic relationship with zooxanthellae (e.g., Reynaud et al., 2003; Anthony et al., 2008; Prada et al., 2017). Yet, future ocean conditions can also have an impact on the survival and growth of other charismatic organisms hosting photosynthetic endosymbionts, such as giant clams (Watson et al., 2012; Watson, 2015) and kleptoplastic sea slugs (Dionísio et al., 2017). Nevertheless, some photosymbiotic organisms have also shown to be resilient to future climate change, including corals (Palumbi et al., 2014) and acoel flatworms (Dupont et al., 2012).

Efficient antioxidant networks and increased levels of stress proteins have been described in autotrophs as protective mechanisms against environmental stress (e.g., Gattuso et al., 1999; Baird et al., 2009). As photosynthesis is a well-known source of reactive oxygen species (ROS), autotrophs must have an efficient antioxidant network to cope with these molecules and maintain high rates of photosynthesis. Despite their harmful potential, photosynthetic ROS are also powerful signaling molecules that are involved in a number of stress related processes, such as growth and developmental stress responses in plants (Foyer and Shigeoka, 2011). The increase in ROS production not only downregulates the activity of photosystem II (PSII) but it also stimulates gene expression, particularly in terms of acclimation and defense mechanisms (Foyer and Shigeoka, 2011). On the other hand, heat shock proteins (HSPs) of chloroplasts have also shown to be important to protect photosynthesis during heat, oxidative and photoinhibitory stress, by defending PSII reaction centers (Nakamoto et al., 2000; Heckathorn et al., 2002; Barua et al., 2003). In photosynthetic symbionts such as corals, HSPs have also proved to play a major role in order to avoid bleaching events (Baird et al., 2009).

In this context, the aim of the present study was to understand the potential effects of short-term (60 days) thermal stress and high CO_2_ levels over one of the most puzzling symbiotic features observed in the animal kingdom: the mollusk-kleptoplast association. The impact of such environmental drivers on tropical (*E. crispata*) and temperate (*E. viridis*) sacoglossan sea slugs bearing kleptoplasts was evaluated considering several endpoints, namely: (i) survival; (ii) photosynthetic efficiency (PSII maximum quantum yield Fv/Fm; relative electron transport rate – relETR); (iii) metabolism (respiration – R; net primary production – NPP); and (iv) oxidative stress response levels (heat shock protein – HSP; GST – glutathione S-transferase – GST).

## Materials and Methods

### Exposure of Adults to Ocean Warming and Acidification

One hundred specimens of the tropical sacoglossan sea slug *E. crispata* (41.1 ± 3.8 mm of total length) were collected off the Florida Keys coastline and shipped to Laboratório Marítimo da Guia (LMG, Cascais, Portugal) by Tropical Marine Centre (TMC, Iberia, Portugal), a marine aquarium wholesaler recognized for its efforts on the sustainable collection and trade of reef organisms and promotion of animal welfare. One hundred and forty-four specimens of the temperate sacoglossan sea slug *E. viridis* (13.3 ± 0.9 mm of total length) were hand collected during low tides, in Cabo Raso (38° 42′ 34.67″ N, 9° 29′ 12.38″ W; Cascais, Portugal).

Upon arrival to the LMG aquatic facilities, organisms were randomly distributed in recirculating life support systems (RAS) according to Dionísio et al. (2013). Each RAS was composed by a 250-L holding aquaria, filled with 0.2 μm altered natural seawater (NSW), and equipped with mechanical (100 μm, TMC Iberia, Portugal), physicochemical (REEF-Skim Pro 400, TMC Iberia, Portugal) and biological (Fernando Ribeiro Lda, Portugal) filtration. All RAS were additionally equipped with UV irradiation (Vecton 600, TMC Iberia, Portugal). Ammonia (<0.5 mg/L) and nitrite (<0.05 mg/L) levels were daily checked using colorimetric test kits (Aquamerk, Merck Millipore, Germany). Overhead tank illumination was provided through dimmable LED illumination apparatus (Aquabeam 1500 Ultima NP Ocean Blue, TMC Iberia, Portugal), consisting of five white XP-G LEDs (9000 K) and five XP-E blue LEDs (50000 K). Photosynthetically active radiation (PAR) was measured (FluorPen FP100 light meter, Photo System Instruments, Czechia) and maintained at 150 ± 15 μmol photons m^−2^ s^−1^ at the water surface, and photoperiod was set to 14 h light: 10 h dark. The siphonaceous macroalgae *Codium tomentosum* and *Bryopsis plumosa* (previously acclimated for 2 days to the same conditions of stocked sea slugs) were provided *ad libitum* as feed source. During the first 2 weeks of laboratory acclimation, sea slugs were kept at control conditions, corresponding to the ambient temperature and pH conditions at collection sites, i.e., 26°C and pH 8.0 for *E. crispata*, and 18°C and pH 8.0 for *E. viridis*). After laboratory acclimation, *E. crispata* individuals were randomly divided into five 5^−L^ tanks per treatment (*n* = 5 individuals per tank, *n* = 25 individuals per treatment), and *E. viridis* into three 5^−L^ tanks per treatment (*n* = 12 individuals per tank, *n* = 36 individuals per treatment). Subsequently, organisms were exposed for 5 days to a gradual increase of *p*CO_2_ and temperature levels. After this period, organisms were exposed for 8 weeks to 4 different experimental conditions, namely: (i) Control scenario – normocapnia (pH 8.0) and control temperature (26 and 18°C for *E. crispata* and *E. viridis*, respectively); (ii) hypercapnia/high CO_2_ scenario – hypercapnia (pH 7.6) and control temperature; (iii) thermal stress scenario (+4°C, i.e., 30 and 22°C for *E. crispata* and *E. viridis*, respectively) and normocapnia; and (iv) thermal stress + high CO_2_ combined scenario – the warming and hypercapnia scenarios.

Seawater temperature and pH were adjusted automatically by using a Profilux control system (GHL, Germany) connected to individual temperature and pH probes (GHL, Germany). The temperature was automatically upregulated by submergible heaters and downregulated using cooling systems (HC-1000A, Hailea, China). Monitoring of pH values was automatically performed (every 2 s) and adjusted via a solenoid valves system, being downregulated through the injection of a certified CO_2_ gas mixture (Air Liquide, Portugal) or upregulated by aerating the tanks with atmospheric filtered air (soda lime, Sigma-Aldrich). Salinity was measured with a refractometer (V2 Refractometer, Tropical Marine Centre, Portugal) and kept at 35 ± 1 μS cm^−1^. Seawater carbonate system speciation (Table 1) was calculated weekly based on total alkalinity (Sarazin et al., 1999), pH, temperature, and salinity measurements using the CO_2_SYS software (Lewis and Wallace, 1998), with dissociation constants accordingly (Mehrbach et al., 1973).

**Table 1:** Seawater carbonate chemistry during the exposure of *E. crispata* and *E. viridis* to different temperature and pH conditions.

Experimental Treatments	Temperature (°C)	pH_T_	A_T_ (μmol kg^−1^ SW)	*p*CO_2_ (μatm)	Ω_aragonite_
*Elysia crispata*					
Control	26.0 ± 0.1	8.0 ± 0.1	2075.9 ± 48.3	393.4 ± 9.6	2.97 ± 0.08
Acidification	26.0 ± 0.1	7.6 ± 0.1	2028.6 ± 37.1	1144.7 ± 21.3	1.30 ± 0.02
Warming	30.0 ± 0.1	8.0 ± 0.1	2063.9 ± 29.1	398.4 ± 5.9	3.30 ± 0.08
Acidification + Warming	30.0 ± 0.1	7.6 ± 0.1	2059.0 ± 27.9	1181.6 ± 16.3	1.51 ± 0.02
*Elysia viridis*					
Control	18.19 ± 0.1	8.0 ± 0.1	2059.1 ± 180.3	466.1 ± 32.4	2.11 ± 0.14
Acidification	18.10 ± 0.1	7.58 ± 0.1	2215.0 ± 88.8	1370.0 ± 55.7	0.97 ± 0.03
Warming	21.95 ± 0.1	8.0 ± 0.1	2058.4 ± 155.2	329.9 ± 26.0	2.86 ± 0.22
Acidification + Warming	21.78 ± 0.2	7.59 ± 0.1	2268.0 ± 125.7	1381.2 ± 77.7	1.16 ± 0.0

*Values for pCO_2_, aragonite saturation state (Ω_aragonite_) were calculated from salinity, temperature, pH total scale (pH_T_) and total alkalinity (A_T_), using CO_2_SYS software (Lewis and Wallace, 1998). Values are represented as mean ± standard deviation (SD; n = 40).*

### Survival and Photo-Physiological Response

Survival at each treatment was daily checked throughout the entire experimental period (i.e., 60 days). The integrity of the symbiosis was evaluated at the initial (*T* = 0), mid (*T* = 30), and final (*T* = 60) days of exposure, based on the presence of green kleptoplasts inside the digestive tubules of the sacoglossan sea slugs. Kleptoplasts were qualitatively evaluated using morphological features, namely color, symmetry and their distribution in the tubules. Images were taken using a binocular microscope (DM1000, Leica, Germany) equipped with a digital camera (DFC 450, Leica, Germany).

Variable chlorophyll *a* fluorescence was measured at day 60 using a PAM (Pulse Amplitude Modulated) fluorometer, comprising a computer-operated PAM-control unit (JUNIOR-PAM, Walz Heinz GmbH, Germany) and a WATER-EDF emitter-detector unit (Gademann Instruments GmbH, Germany). The actinic and saturating light was provided by a blue LED-lamp (450 nm peak and 20 nm half-band width) and supplied through a plastic fiber optic bundle (1.5 mm diameter) perpendicularly positioned to the surface of the sea slug parapodia. A saturation pulse of 2500 μmol photons m^−2^ s^−1^ with a duration of 0.8 s was applied to at least 8 slugs per treatment (previously anaesthetized as described in Cruz et al. (2012), in order to determine the fluorescence at both dark and light conditions. Sea slugs were dark-adapted for 30 min and the minimum (*F*_o_) and maximum fluorescence (*F*_m_) in the dark-adapted state were used to determine the variable fluorescence (*F*_v_ = *F*_m_ – *F*_o_) and the maximum quantum yield of PSII (*F*_v_/*F*_m_). Sea slugs were then light-adapted at 150 ± 15 μmol photons m^−2^ s^−1^ for 30 min. The minimum (F) and maximum fluorescence (*F*_m_’) in the light-adapted state were used to determine the variable fluorescence (**Δ***F* = *F*_m_’ – *F*) and the PSII maximum quantum yield (**Δ***F*/*F*_m_’) in the light-adapted state. The relETR was then calculated as:

$$\text{relETR}=\Delta F/F{\text{m}}^{\prime }\times \text{PAR}\times 0.5$$

where PAR is the photosynthetic active radiation and 0.5 compensates for irradiance being split between two photosystems.

### Sea Slug Metabolism

Oxygen consumption was determined 60 days after exposure to experimental scenarios according to previously established methods (Rosa et al., 2009, 2012, 2013). Sea slugs (*n* = 6 per treatment) were individually incubated in sealed water-jacketed respirometry chambers (Strathkelvin, United Kingdom) containing 1 μm filtered and UV-irradiated NSW derived from the respective experimental treatments. Water volumes were adjusted in relation to animal mass (up to 3 mL) in order to minimize locomotion and stress but still allow for spontaneous and routine activity rates. Respiration chambers were immersed in Lauda water baths (Lauda-Brinkmann, Germany) to control temperature. Oxygen concentrations were recorded with Clark-type O_2_ electrodes connected to a multi-channel oxygen interface (Model 928, Strathkelvin, United Kingdom). Controls (blanks) were used to correct for possible bacterial respiratory activity. Two runs of 3 h were made per individual, one exposed to light and the other in complete darkness to inhibit photosynthesis. Light or dark incubations were performed within the respective photoperiod of the animals. Oxygen concentration measurements (μmol O_2_ L^−1^) were transformed into μmol O_2_ g^−1^ L^−1^ h^−1^ by taking into consideration the volume of the chamber and the wet weight of the slug. Respiration was determined as the oxygen consumption rate in complete darkness, while net primary photosynthesis was determined as the oxygen production rate in the light exposed conditions, according to Baker et al. (2015).

### Oxidative Stress Response of Sea Slug

The oxidative stress response was analyzed based on both the HSP production (HSP70/HSC70) and the activity of the antioxidant enzyme (GST). A total of 3 samples (each one containing 3 slugs) were analyzed per treatment. Samples were homogenized using an Ultra-Turrax (Staufen, Germany) in phosphate-buffered saline (PBS), pH 7.4: 0.14 M NaCl (≥99%), 2.7 mM KCl (≥99%), 8.1 mM Na_2_HPO_4_ (≥99%), and 1.47 mM KH_2_PO_4_ (≥99%), Sigma-Aldrich, United States) and centrifuged at 10,000 ×*g* for 15 min at 4°C. Afterward, homogenized samples were frozen at -80°C until further analyses.

Total protein measurements were determined according to Bradford (1976) adapted to 96-well microplates. Briefly, 20 μL of each sample and 200 μL of 5 % Bradford reagent solution (Sigma-Aldrich, United States) were added to a 96-well microplate and the absorbance read at 595 nm (Asys UVM 340, Biochrom, United States). Albumin bovine serum (BSA, Sigma-Aldrich, United States) dilutions (0–1 mg) were used as standards. Bradford results were then used to normalize HSP and GST results to total protein content.

The HSP70/HSC70 content was assessed by Enzyme-Linked Immunosorbent Assay (ELISA), by adapting the protocol from Njemini et al. (2005) (see more details in Supplementary Methods). Briefly, 10 μL of the homogenate supernatant was diluted in 250 μL of PBS. Afterward, 50 μL of the diluted sample was added to 96-well microplates (Nunc- Roskilde, Denmark) and allowed to incubate overnight at 4°C. After 24 h, the microplates were washed in PBS containing 0.05% Tween-20 (≥40%, Sigma-Aldrich, United States). A total of 100 μL of blocking solution (1% bovine serum albumin, Sigma-Aldrich, United States) was added to each well and left to incubate at room temperature for 2 h. After washing the microplates, 50 μL of a solution of 5 μg mL^−1^ of primary antibody (anti-HSP70/HSC70, Acris, United States) was added to each well and then incubated at 37°C for 90 min. According to the manufacturer details, the primary antibody (anti-HSP70/HSC70) has a broad range of reactivity. The primary antibody reactivity for the species *E. crispata* and *E. viridis* was validated by Western blot. The non-linked antibody was removed by an additional washing step of the microplates. The alkaline phosphatase-conjugated anti-mouse IgG (Fab specific, Sigma-Aldrich, United States) was then used as a secondary antibody, by adding 50 μL of a solution at 1 μg mL-1 to each well and incubating the microplates for 90 min at 37°C. After three additional washing steps, 100 μL of substrate (SIGMA*FAST*^TM^ *p*-nitrophenyl phosphate tablets, Sigma-Aldrich, United States) was added to each well and incubated for 10–30 min at room temperature. Subsequently, 50 μL of stop solution (3 M NaOH (≥98%), Sigma-Aldrich, United States) was added to each well, and the absorbance was read at 405 nm in a 96-well microplate reader (Asys UVM 340, Biochrom, United States). The concentration of HSP70/HSC70 in the samples was calculated from a curve of absorbance based on serial dilutions (between 0 and 2 μg mL^−1^) of purified HSP70 active protein (Acris, United States). Results were expressed in relation to the protein content of the samples, which was determined according to Bradford (1976).

The activity of the antioxidant enzyme GST was determined according to Rosa et al. (2012) and Lopes et al. (2013) and optimized for a 96-well microplate. This assay uses 1-chloro-2,4-dinitrobenzene (CDNB) as substrate, which conjugates with the thiol group of the glutathione (GSH) causing an increase in absorbance. A total of 180 μL of substrate solution (composed by 200 mM L-glutathione reduced in Dulbecco’s PBS and 100 mM CDNB (≥99%)) was added to each well of a 96-well Nunclon microplate (Thermo Scientific Nunc, ıUnited States), along with 20 μL of GST standard (≥25 units/mg protein, Sigma-Aldrich, United States) or sample. Equine liver GST was used as a positive control to validate the assay. The enzyme activity was determined spectrophotometrically at 340 nm by measuring the formation of the conjugate of GSH (≥99%, Sigma-Aldrich) and CDNB (≥99%, Sigma-Aldrich). The absorbance was recorded every minute for 6 min, using a plate reader (BioRad, United States). The increase in absorbance per minute was estimated and the reaction rate at 340 nm was determined using the CDNB extinction coefficient of 0.0053*ϵ*μM, as follows:

$$\mathrm{GST activity}=\frac{\Delta \text{A}340/\min}{0.0053}\times \frac{\text{TV}}{\text{SV}}\times \text{DF}$$

where TV is the total volume, ST is the sample volume and DF is the dilution factor. Results were expressed in relation to the protein content of the samples, which was determined according to the Bradford method (Bradford, 1976).

### Statistical Analysis

All data were analyzed using generalized linear mixed models (Zuur et al., 2009). The distributional family used was Binomial (logit link function) for proportions (i.e., survival), Gaussian (identity link function) for quantities (i.e., F_v_/F_m_ and relETR), and Gamma (log link function) for positive quantities with a severe positively skewed distribution (i.e., R and NPP). The sample size of oxidative stress variables was not enough to model HSP and GST as response variables, which were therefore analzsed only through descriptive statistics. The initial mixed models included the species, temperature and pH as fixed effects, the corresponding second and third order interactions, and the tank as a random effect to account for possible dependency within tanks. Following the recommendation from Barr et al. (2013), the random effects were kept in the models irrespectively of the amount of variation they explained.

The most parsimonious models were selected based on the Akaike Information Criterion. Model residuals were checked for departures from the assumed distributions and no significant deviations were found. For Binomial models, odds ratios and confidence limits were determined to allow a more informative discussion of the results. Considering that odds define the ratio of the probability of success and the probability of failure, odds ratios were built by the ratio of odds between the two species (*E. crispata* vs. *E. viridis*), temperatures (control temperature vs. warming) or pH (normocapnia vs. hypercapnia).

All statistical analyses were implemented in R, using the lme4 (Bates et al., 2015) and nlme (Pinheiro et al., 2018) packages. Results were considered statistically significant at a significance level of 0.05.

## Results

### Survival

**FIGURE 1:**
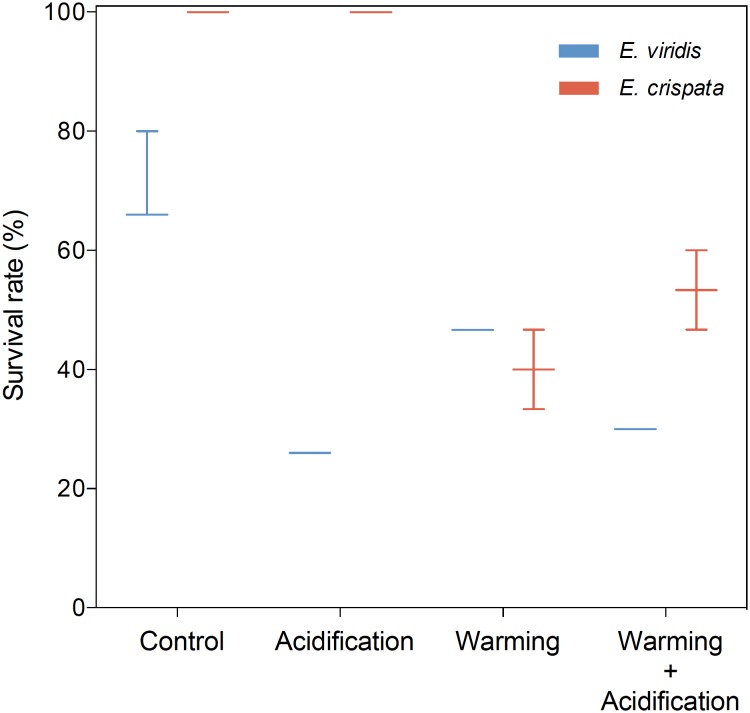
Effects of ocean warming and acidification on survival (%) of tropical *E. crispata* and temperate *E. viridis* species, under different climate change scenarios, i.e., control (18 and 26°C, pH8.0); acidification (18 and 26°C, pH7.6); warming (22 and 30°C, pH8.0) and acidification + warming (22 and 30°C, pH7.6) experimental treatments. Tukey box-plots show median, percentile 25th and 75th, and –1.5 times interquartile distance (IQR) and +1.5 times IQR, respectively.

Sea slug survival was significantly affected by temperature (*p* = 0.005) but not by high CO_2_ (*p* = 0.624) (Figure 1). The odds of survival under control temperature were more than “7 times higher” than the odds of survival under warming conditions. No mortality was observed under control conditions for both species. *E. crispata* survival decreased under warming conditions to 40 ± 34.6 and 53.3 ± 30.6% under normocapnia and acidification, respectively. *E. viridis* survival decreased under warming conditions down to 72.2 ± 9.6 and 41.7 ± 8.3%, under normocapnia and acidification, respectively. Moreover, no significant differences were found between species (*p* = 0.152), although the interaction between species and pH was found to be significant (*p* = 0.004). While we cannot detect a pH effect over *E. crispata* survival, *E. viridis* survival decreased under hypercapnia by 69.4 and 30.6 percentage points, under control temperature and heat conditions, respectively (see more statistical details in Supplementary Table S1).

### Photo-Physiological Responses

**FIGURE 2:**
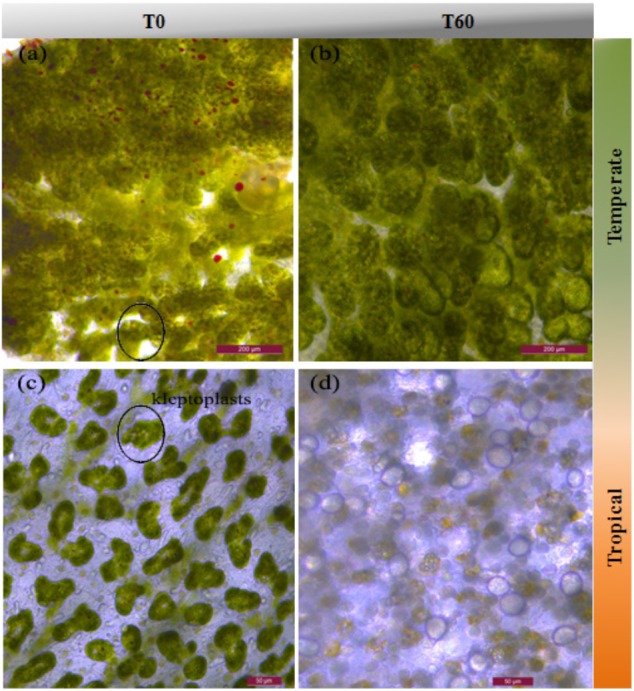
Light micrographs of the termini of the digestive diverticula tubules of *E. viridis* **(a,b)** and *E. crispata*. **(a)** Kleptoplasts of *E. viridis* at T0 (Control, 18°C, pH8.0). Kleptoplasts are packed tightly in the tubule cells and ramify throughout the body. **(b)** Kleptoplasts of *E. viridis* at T60 (Acidification + Warming, 22°C, pH7.6); **(c)** kleptoplasts of *E. crispata* at T0 (Control, 26°C, pH8.0). The main area is traversed by small digestive diverticula; kleptoplasts are located along the length of the tubules as well in the tip of the tubule (black circle) (Curtis, 2006). **(d)** Bleaching of *E. crispata* kleptoplasts at T60 – (Acidification + Warming, 30°C, pH7.6). Scale bar: **(a,b)** 200 μm and **(c,d)** 50 μm.

Under control conditions, *E. viridis*’ kleptoplasts were packed tightly in the tubule cells surrounding the terminus of the tubule (Figure 2a). Neither high temperature nor high CO_2_ affected the color or the morphology of kleptoplasts (Figure 2b). In contrast, *E. crispata* kleptoplasts were mainly distributed in the tip of the tubule cells under control conditions (Figure 2c). Bleaching was observed in all the slugs exposed to warming condition, with the majority of the host tubule cells being unfilled or displaying degraded kleptoplasts (Figure 2d).

**FIGURE 3:**
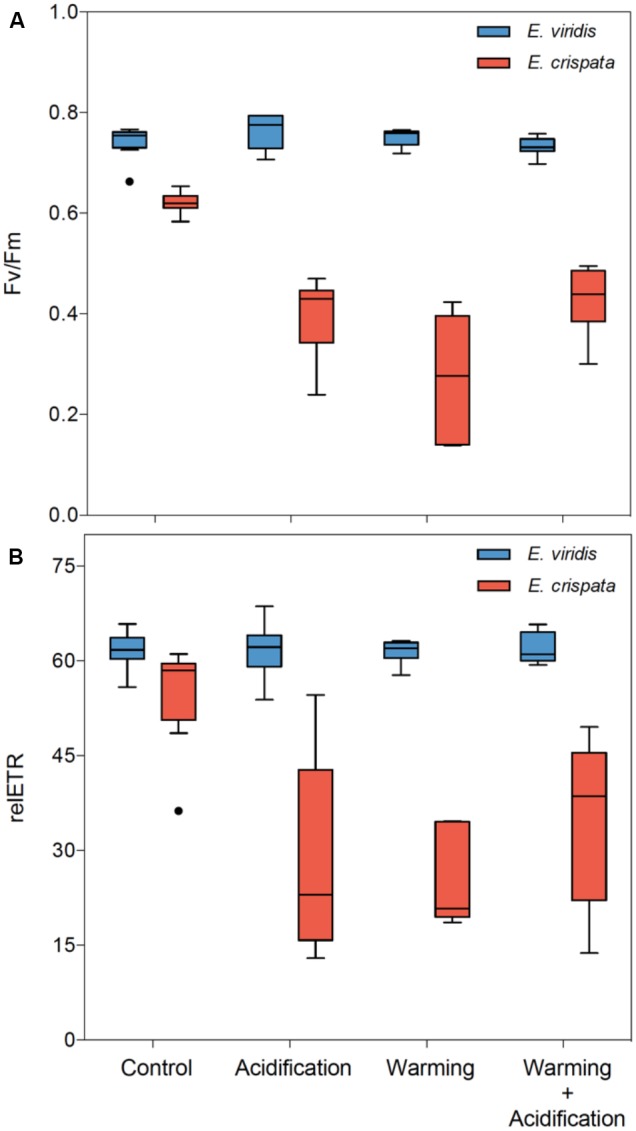
Effects of ocean warming and acidification on the photobiology of kleptoplasts within tropical *E. crispata* and temperate *E. viridis* species. **(A)** F_v_/F_m_, and **(B)** relETR under different climate change scenarios, i.e., control (18 and 26°C, pH8.0); acidification (18 and 26°C, pH7.6); warming (22 and 30°C, pH8.0) and acidification + warming (22 and 30°C, pH7.6) experimental treatments. Tukey box-plots show median, percentile 25th and 75th, and **–**1.5 times IQR and **+**1.5 times IQR, respectively.

The photosynthetic efficiency of kleptoplasts (Figure 3) was significantly affected by temperature (*p* < 0.001 for F_v_/F_m_ and relETR) and pH (*p* = 0.026 for F_v_/F_m_ and *p* = 0.001 for relETR), but these effects varied between species (*p* < 0.001 for F_v_/F_m_ and *p* = 0.003 for relETR). Moreover, the interaction between species and temperature was also significant (*p* = 0.022 for F_v_/F_m_ and *p* = 0.030 for relETR). While the F_v_/F_m_ and relETR of *E. viridis* varied little among treatments, the photosynthetic efficiency of *E. crispata* decreased under both hypercapnia and heat conditions. More specifically, F_v_/F_m_ decreased 35.9 and 55.3%, while relETR decreased 48.9 and 53.0% under hypercapnia and heat, respectively. However, under the combined effect of hypercapnia and heat, the negative impact of these variables was not cumulative, resulting in a significant interaction between temperature and pH (*p* = 0.012 for F_v_/F_m_ and *p* = 0.005 for relETR; see more statistical details in Supplementary Table S1).

### Metabolism

**FIGURE 4:**
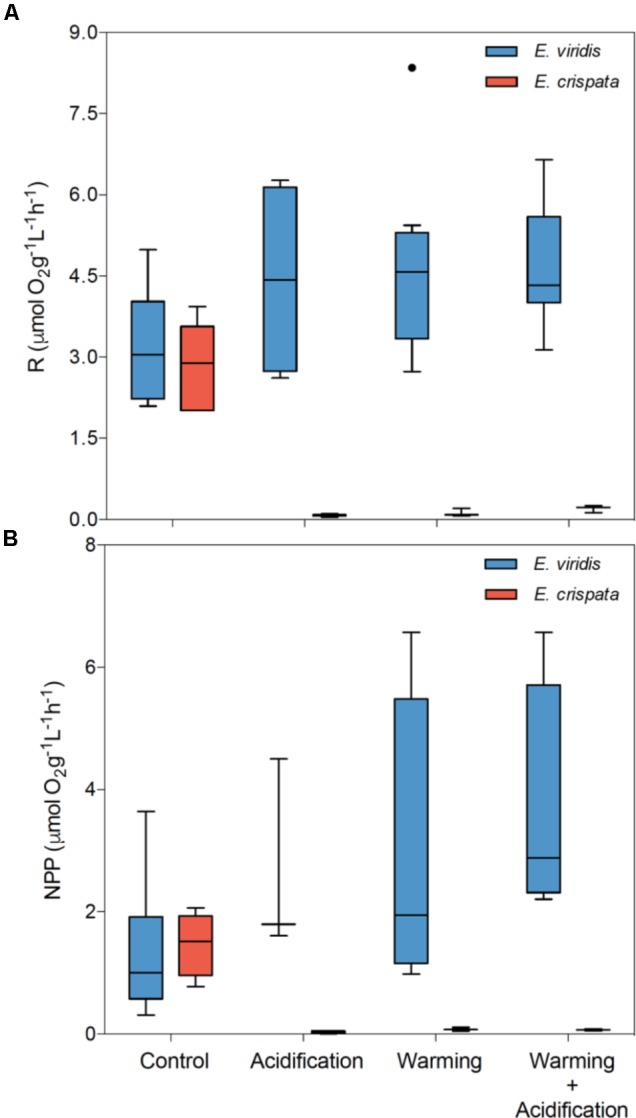
Effects of ocean warming and acidification on the tropical *E. crispata* and the temperate *E. viridis* species. **(A)** R – respiration and **(B)** NPP – net primary production under different climate change, i.e., control (18 and 26°C, pH8.0); acidification (18 and 26°C, pH7.6); warming (22 and 30°C, pH8.0) and acidification + warming (22 and 30°C, pH7.6) experimental treatments. Tukey box-plots show median, percentile 25th and 75th, and **–**1.5 times IQR and **+**1.5 times IQR, respectively.

Sea slug metabolism was significantly affected by pH (*p* < 0.001), but not by temperature (*p* = 0.673) (Figure 4). Moreover, the interaction between species and pH was also significant (*p* < 0.001 for R and NPP). NPP was significantly affected by both pH (*p* < 0.001) and temperature (*p* = 0.043). Moreover, the interaction between species and pH (*p* < 0.001) and between species and temperature (*p* = 0.045) was also significant (see more statistical details in Supplementary Table S1). While *E. viridis* metabolism varied little or even increased, the metabolism of *E. crispata* decreased to values near zero under acidification and/or warming conditions.

### Oxidative Stress Response

**FIGURE 5:**
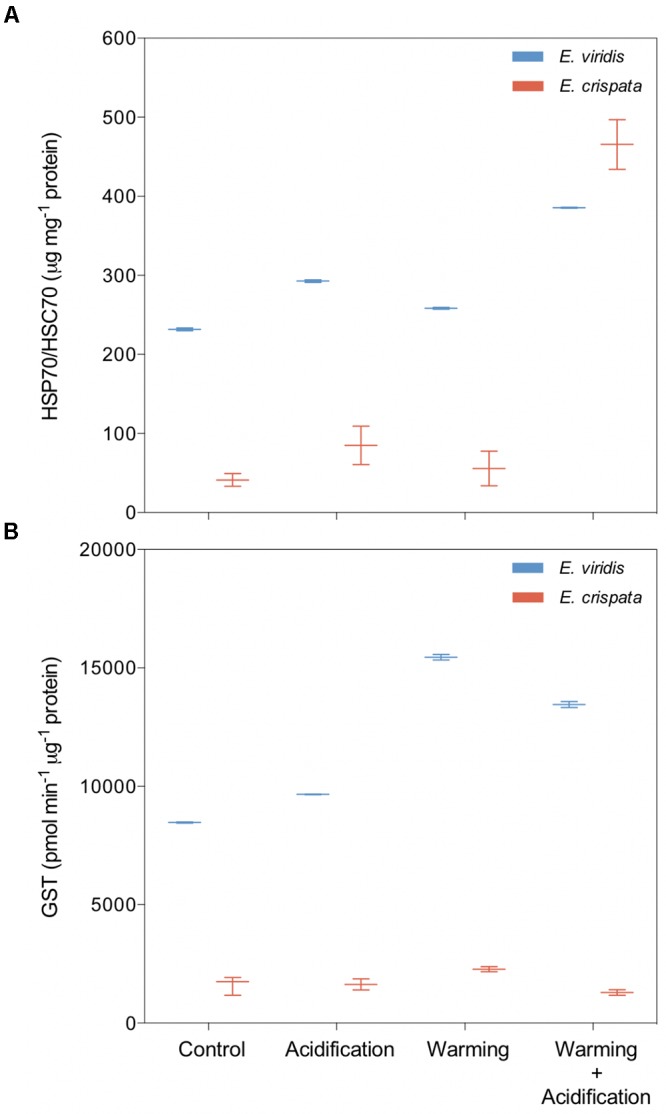
Effects of ocean warming and acidification on the heat shock response (HSP) and antioxidant defense (GST) of tropical *E. crispata* and the temperate *E. viridis*. **(A)** HSP and **(B)** GST, under different climate change scenarios, i.e., control (18 and 26°C, pH8.0); acidification (18 and 26°C, pH7.6); warming (22 and 30°C, pH8.0) and acidification + warming (22 and 30°C, pH7.6) experimental treatments. Tukey box-plots show median, percentile 25th and 75th, and **–**1.5 times IQR and **+**1.5 times IQR, respectively.

*Elysia viridis* revealed significantly higher HSP content than *E. crispata* (Figure 5A). However, when exposed to the combined scenario, *E. crispata* showed the highest value observed (418.6 μg HSP70/mg of total protein. In *E. viridis*, such increase was from 232.4 to 385.3 μg HSP70/mg of total protein. Besides the recurrent interspecific differences, GST levels increased 58.8% with warming in *E. crispata* (Figure 5B); yet, such response was not observed under the combination of both stressors (with a down-regulation of 20% compared to control treatment. Regarding *E. viridis*, GST levels were significantly higher under both warming and warming+acidification treatments.

## Discussion

Certain habitats are subject to rapid fluctuations in physical characteristics across tidal cycles, where coastal sea slugs (such as the present studied species) can be submitted to aerial emersion, thermal stress and desiccation. Such exposition is known to significantly affect organisms’ physiological state, survival and growth (Dong et al., 2008; Teixeira et al., 2013). Although aware of the limitation of the present experimental design (i.e., stable environmental conditions throughout the entire acclimation period), the present findings seem to corroborate, at a first glance, the idea that marine tropical biota are expected to be more sensitive to warming than temperate organisms, as they evolved in a relatively stable thermal environment (Tewksbury et al., 2008; Nilsson et al., 2009; Rosa et al., 2014). However, it is worth noting that, even though kleptoplasty in the temperate species was not impaired, the same was not observed for survival. When compared to control conditions, *E. viridis* survival decreased 58.3% under the combined effect of heat and high CO_2_. High temperature was the main factor affecting the survival of both species, while pH only affected the survival of the temperate model (i.e., elevated *p*CO_2_ *per se* did not influence the survival of *E. crispata*). Thus, we argue that the tropical (stenotherm) sea slug species may display a greater scope for acclimatization than the temperate (eurytherm) counterpart (see also Verberk et al., 2016, and references therein). In fact, intertidal species such as *E. viridis* are exposed to a wide and higher range of daily pH fluctuations, tolerating pH values as low as 7.4 or even lower during night time, when photosynthesis does not occur and CO_2_ from respiration accumulates in tidal pools (Cornwall et al., 2013). The present study shows that *E. viridis* was unable to survive under long-term exposure to high *p*CO_2_ conditions. Several studies have shown that stenotherms, such as tropical organisms, may rise their fitness in a narrower thermal niche and concomitantly minimize maintenance costs (Dillon et al., 2010; Fusi et al., 2014; Seebacher et al., 2015). Thus, thermal specialists, such as *E. crispata*, may have a larger scope for acclimatization than eurytherms, like *E. viridis*. Such advantage may turn stenotherms less vulnerable to environmental warming because they can display niche shifts through both plastic responses or rapid evolution (Hoffmann and Sgrò, 2011; van Heerwaarden and Sgrò, 2014; Verberk et al., 2016).

The integrity of the symbiosis displayed between sacoglossan sea slugs and their kleptoplasts was not identical considering the studied specimens (i.e., temperate and tropical species). While in *E. viridis* the mollusk-plastid association remained stable under the combined treatment, high temperature led to chloroplast degradation and bleaching in *E. crispata*. This phenomena, i.e., the disruption of the symbiotic association, has already been recorded in cnidarian tropical species (Gates et al., 1992; Fitt et al., 2001), a scenario which is aggravated under the combined effect of heat and hypercapnia (Rodolfo-Metalpa et al., 2011; Kroeker et al., 2013; Kaniewska et al., 2015). In accordance, the photosynthetic efficiency of the tropical symbionts being hosted has also shown to decrease under heat stress. While the F_v_/F_m_ and relETR remained stable in *E. viridis* under the different tested climate change scenarios, the kleptoplasts hosted by *E. crispata* showed a marked decrease in both photobiological parameters monitored under thermal challenges and hypercapnia. The rates of respiration and photosynthesis slightly varied (increased) in the case of *E. viridis* exposed to heat and high *p*CO_2_ conditions, which indicates that this species may be capable of displaying a high photosynthetic performance even under such harsh environmental conditions. Nonetheless, as in corals with *Symbiodinium*, the expulsion of kleptoplasts by the tropical sea slug is a physiological response to environmental stress that does not necessarily imply signs of vulnerability in the host itself.

Enhanced rates of photosynthesis and respiration have been observed in temperate sea anemones and corals exposed to elevated *p*CO_2_ (Crawley et al., 2010; Suggett et al., 2012; Towanda and Thuesen, 2012; Gibbin et al., 2014). In contrast, both R and NPP decreased significantly down to values near zero in specimens of *E. crispata*, subject to heat and/or hypercapnic conditions. Metabolic depression is a widespread strategy to withstand environmental stress that is characterized by the shutting down of expensive processes to save energy and ensure long-term survival. Under control conditions, *E. crispata* presented lower HSP and GST levels than *E. viridis*. This finding is not surprising as intertidal organisms that experience highly variable thermal conditions (such as *E. viridis*) activate their heat shock response more frequently to withstand thermal fluctuations (Lesser, 2006). In contrast, marine organisms occupying stable thermal environments (such as the tropical species *E. crispata*) do not need to cope with thermal fluctuations and may even lack a heat shock response (Tomanek, 2008). Similarly, basal GST levels of *E. crispata* were also lower than those of *E. viridis*. Considering HSP and antioxidant response, they were enhanced under heat and high CO_2_. HSP levels increased in both species (1.7 and 11.3 times in *E. viridis* and *E. crispata*, respectively) under the combined scenario. Although *E. crispata* presented lower basal HSP levels than *E. viridis*, its response to environmental stress was much more pronounced. Increased expression of HSPs protects the cells against protein unfolding and damage due to environmental stress (Tomanek, 2008) and has been observed in other photosymbionts exposed to warming and acidification (Heckathorn et al., 2004; Moya et al., 2015).

The mean values recorded for GST increased in *E. viridis* exposed to these environmental disturbances, especially under heat conditions. In contrast, *E. crispata* showed a poor antioxidant defense capacity. These results are in line with previously reported ones, for the tropical sacoglossan sea slug *E. cornigera* (de Vries et al., 2015). Indeed tropical species appear to accumulate ROS in a much higher degree than the temperate *E. timida*, thus suggesting a potential dichotomy in antioxidant capacities between tropical and temperate species. Our results, along with the reduced photosynthetic efficiency of tropical *E. crispata* under heat and high CO_2_ conditions, suggest that heat shock and antioxidant response may play an important role as mechanisms for stabilizing photosynthesis under stress conditions, a feature already reported for tropical reef forming corals hosting photosymbionts (Bhagooli and Hidaka, 2004; Moya et al., 2015). Last, it is worth noting that these findings (namely metabolic and HSP/antioxidant data) should be looked with cautious because they were obtained from a complex system of host-symbiont interaction, and not in the single organism.

Overall, our results revealed that the mollusk-plastid associations in temperate habitats seems to be more vulnerable to heat stress and hypercapnia in comparison to tropical ones. While the temperate *E. viridis* showed photo-physiological tolerance (i.e., absence of bleaching), its survival was the most negatively affected. Thus, we argue that *E. crispata* may exhibit increased capacity for phenotypic plasticity and acclimation responses in comparison to *E. viridis* (see also Fusi et al., 2014), and may potentially face harsh environmental conditions more effectively than their generalist counterparts (see also Dillon et al., 2010; Seebacher et al., 2015; Verberk et al., 2016). Thus, two issues are certainly worth investigating in future studies: (1) could the physical and biochemical feed properties be influenced by abiotic aquatic parameters and subsequently lead to biological backlashes (e.g., starvation, bleaching, survival) in sacoglossan sea slug? and (2) is such high vulnerability to future temperate conditions also displayed by other species exhibiting functional mollusk-plastid associations?

## Ethics Statement

Research was conducted under approval of Faculdade de Ciências da Universidade de Lisboa animal welfare body (ORBEA) and Direção-Geral de Alimentação e Veterinária (DGAV) in accordance with the requirements imposed by the Directive 2010/63/EU of the European Parliament and of the Council of 22 September 2010 on the protection of animals used for scientific purposes.

## Author Contributions

GD, RR, and RC designed the experiments. GD and ARL performed the experiments. GD, FF, TR, SC, RB, JRP, and RR analyzed the data. All authors contributed to the writing of the manuscript.

## Conflict of Interest Statement

RB was employed by company Startfactor. The remaining authors declare that the research was conducted in the absence of any commercial or financial relationships that could be construed as a potential conflict of interest.
