# Supplementary material for: Distinct Bleaching Resilience of Photosynthetic Plastid-Bearing Mollusks Under Thermal Stress and High CO2 Conditions
Source: Front Physiol. 2018 Nov 30;9:1675. doi: 10.3389/fphys.2018.01675 (PMC6284066; doi:10.3389/fphys.2018.01675)
Supplement: Supplementary file 1 [file Table_1.docx]

**Supplemental Material**

**TABLE S1. Generalized Linear Mixed Models (GLMM) analysis of the physiological variables measured in two sea slug species (*E. viridis* and *E*. *crispata*). Distribution family, response variables, initial and final model terms and random effects are presented on top of each model. Bold values indicate p < 0.05.**

| *Model: GLMM (Binomial)* | | ***Response variable: Survival*** | | |
| --- | --- | --- | --- | --- |
| *Initial model terms: CO_2,_ temperature, species and two-order interactions between them* | | |  |  |
| *Final model terms: CO_2,_ temperature, species and interaction between CO_2_ and temperature* | | |  |  |
| *Random factor: Tank ID* | Estimate | Std. Error | z value | p-value |
| (Intercept) | 2.305 | 0.853 | 2.701 | **0.007** |
| Species | 1.228 | 0.858 | 1.431 | 0.152 |
| Warming | -2.056 | 0.732 | -2.81 | **0.005** |
| High CO_2_ | 0.446 | 0.91 | 0.49 | 0.624 |
| Warming × High CO2 | -3.565 | 1.228 | -2.903 | **0.004** |
| *Model: GLMM (Gaussian)* | | ***Response variable: Fv/Fm*** | |  |
| *Initial model terms: CO_2,_ temperature, species and two-order interactions between them* | | | | |
| *Final model terms: CO_2,_ temperature, species and interaction between CO_2_ and temperature and between species and temperature* | | | | |
| *Random factor: Tank ID* | Estimate | Std. Error | t value | p-value |
| (Intercept) | 0.558 | 0.035 | 15.943 | **<0.001** |
| Species | 0.244 | 0.04 | 6.114 | **<0.001** |
| Warming | -0.231 | 0.049 | -4.691 | **<0.001** |
| High CO_2_ | -0.097 | 0.04 | -2.428 | **0.026** |
| Warming × Species | 0.141 | 0.056 | 2.508 | **0.022** |
| Warming × High CO2 | 0.156 | 0.056 | 2.778 | **0.012** |
| *Model: GLMM (Gaussian)* | | ***Response variable: ETR*** | |  |
| *Initial model terms: CO_2,_ temperature, species and two-order interactions between them* | | | | |
| *Final model terms: Same as the initial model* | | | | |
| *Random factor: Tank ID* | Estimate | Std. Error | t value | p-value |
| (Intercept) | 51.746 | 2.928 | 17.675 | **<0.001** |
| Species | 13.661 | 3.985 | 3.428 | **0.003** |
| Warming | -19.334 | 4.467 | -4.328 | **0.001** |
| High CO_2_ | -17.364 | 4.438 | -3.913 | **0.001** |
| Warming × Species | 11.899 | 5.029 | 2.366 | **0.03** |
| Species × High CO2 | 9.239 | 5.039 | 1.834 | **0.084** |
| Warming × High CO2 | 16.292 | 5.009 | 3.253 | **0.004** |
| *Model: GLMM (Gamma)* | | ***Response variable: Respiration*** | | |
| *Initial model terms: CO_2,_ temperature, species and two-order interactions between them* | | | | |
| *Final model terms: CO_2,_ temperature, species and interaction between species and CO_2_ and between species and temperature* | | | | |
| *Random factor: Tank ID* | Estimate | Std. Error | t value | p-value |
| (Intercept) | 0.675 | 0.334 | 2.021 | **0.043** |
| Species | 0.567 | 0.494 | 1.147 | 0.241 |
| Warming | -0.278 | 0.658 | -0.422 | 0.673 |
| High CO_2_ | -2.655 | 0.65 | -4.279 | **<0.001** |
| Warming × Species | 0.518 | 0.774 | 0.668 | 0.504 |
| Species × High CO2 | 2.793 | 0.74 | 3.775 | **<0.001** |
| *Model: GLMM (Gamma)* |  | ***Response variable: NPP*** | |  |
| *Initial model terms: CO_2,_ temperature, species and two-order interactions between them* | | |  |  |
| *Final model terms: CO_2,_ temperature, species and interaction between species and CO_2_ and between species and temperature* | | | | |
| *Random factor: Tank ID* | Estimate | Std. Error | t value | p-value |
| (Intercept) | -0.101 | 0.381 | -0.285 | 0.776 |
| Species | 0.47 | 0.54 | 0.87 | 0.384 |
| Warming | -0.581 | 0.685 | -0.848 | 0.396 |
| High CO_2_ | -2.598 | 0.673 | -3.861 | **<0.001** |
| Warming × Species | 1.164 | 0.841 | 1.384 | 0.166 |
| Species × High CO2 | 3.064 | 0.835 | 3.672 | **<0.001** |
