# Supplementary material for: Distinct Bleaching Resilience of Photosynthetic Plastid-Bearing Mollusks Under Thermal Stress and High CO2 Conditions
Source: Front Physiol. 2018 Nov 30;9:1675. doi: 10.3389/fphys.2018.01675 (PMC6284066; doi:10.3389/fphys.2018.01675)
Supplement: Supplementary file 2 [file Data_Sheet_1.docx]

**Supplementary Methods**

To validate ELISA, here we followed the guidelines described in [1,2], more specifically:

1) When performing an ELISA, with a new antibody, for the first time, we determine the spike recovery to evaluate the effect that sample constituents have on detection of the antigen by the antibody.

2) We always use ELISA samples in duplicate or triplicate, providing enough data for statistical validation of the results. The average absorbance values for each set of duplicates (or triplicate) standards and duplicate (or triplicate) samples should be within 20% of the mean. However, in our laboratory, most of the times, we restrict this value to 10% of the mean.

3) We often use a known sample as positive control. The concentration of the positive control sample should be within the linear section of the standard curve to obtain valid and accurate results.

4) In addition, Blanks NSB (non-specific binding), B0 (maximum binding in absence of the analyte), Standards, Controls (positive and negative), normalization report, control report are analysed or used as routine using the Bio-RAD Microplate Manager Software v4.0 or above or an equivalent software.

5) In addition to standard deviation (SD), we use the coefficient of variation, which is the ratio of the standard deviation σ to the mean μ and express it as a percentage of variance to the mean (%CV). This indicates us any inconsistencies and inaccuracies in the results. Usually, we only accept results if %CV is lower than 10%.

6) We also calculate LOD and LOQ and assay precision as described by [3,4].

For instance, for the current assay LOD was calculated as LOD=0.15 µg/mL and LOQ= 1.5 µg/mL. However, LOD and LOQ calculations it may depend in each ELISA according to assay conditions (e.g. antibodies used, standards, etc.) and standard curves results.

References

[1] ABCAM (2018). Calculating and evaluating ELISA data. https://www.abcam.com/protocols/calculating-and-evaluating-elisa-data

[2] Andreasson U. et al. (2015). A Practical Guide to Immunoassay Method Validation . Frontiers in Neurology , vol. 6, 179. DOI: 10.3389/fneur.2015.00179

[3]Hayashi Y., Matsuda R., Maitani T (2004). Precision, Limit of Detection and Range of Quantitation in Competitive ELISA Anal. Chem., 76, 1295-1301

[4] Shrivastava A, Gupta VB. Methods for the determination of limit of detection and limit of quantitation of the analytical methods. Chron Young Sci 2011;2:21-5.
